# Gonadotropin-Dependent Precocious Puberty: Neoplastic Causes and Endocrine Considerations

**DOI:** 10.1155/2011/184502

**Published:** 2011-01-27

**Authors:** Matthew D Stephen, Peter E Zage, Steven G Waguespack

**Affiliations:** 1 Department of Pediatrics, The University of Texas Health Science Center, Houston, TX 77030, USA; 2 Department of Pediatrics-Patient Care, The University of Texas MD Anderson Cancer Center, Houston, TX 77030, USA; 3 Department of Endocrine Neoplasia and Hormonal Disorders, The University of Texas MD Anderson Cancer Center, 1400 Pressler Street, Unit 1461, Houston, TX 77030-3722, USA

## Abstract

Premature activation of the hypothalamic-pituitary-gonadal (HPG) axis manifests as gonadotropin-dependent precocious puberty. The mechanisms behind HPG activation are complex and a clear etiology for early activation is often not elucidated. Though collectively uncommon, the neoplastic and developmental causes of gonadotropin-dependent precocious puberty are very important to consider, as a delay in diagnosis may lead to adverse patient outcomes. The intent of the current paper is to review the neoplastic and developmental causes of gonadotropin-dependent precocious puberty. We discuss the common CNS lesions and human chorionic gonadotropin-secreting tumors that cause sexual precocity, review the relationship between therapeutic radiation and gonadotropin-dependent precocious puberty, and finally, provide an overview of the therapies available for height preservation in this unique patient population.

## 1. Introduction

The onset of isosexual puberty is typically heralded by breast development in girls and testicular enlargement in boys, usually followed by pubarche/adrenarche, the pubertal growth spurt, and completion of secondary sexual development. Traditionally, normal pubertal onset is considered to occur between 8 and 13 years in girls and between 9 years 6 months and 13 years 6 months in boys [1, 2]. Recent data suggests that pubertal onset is occurring at earlier ages in girls, especially among ethnic minorities and those with higher body mass indices [3–10]. Therefore, it has been suggested to redefine the age of precocious puberty in non-Hispanic black girls to <6 years of age and to <7 years in all other girls [8]. It remains generally accepted that pubertal onset at less than 9 years remains precocious in boys. Significant controversy has risen from these recommendations, given the possible risk of delaying or missing the diagnosis of a pathologic cause of precocious puberty [11, 12]. Importantly, sexual development that occurs at a very young age or puberty that progresses asynchronously or at an accelerated tempo may indicate underlying pathology. 

The prevalence of precocious puberty has been estimated to be at least 10–20-fold higher in girls compared with boys [13]. However, the likelihood of finding an organic cause of precocious puberty is much higher in boys than girls [13–16]. Neoplastic causes of precocious puberty are uncommon but nonetheless important etiologies of precocious sexual development, and prompt recognition of these rare presentations is paramount. 

The intent of the current manuscript is to review the neoplastic and developmental causes of gonadotropin-dependent precocious puberty and to share some of our clinical experience at the Children's Cancer Hospital of the University of Texas M D Anderson Cancer Center. We will not review gonadotropin-independent sexual precocity, such as seen with sex steroid production by primary adrenal or gonadal neoplasms. We will also discuss the potential effects of radiation therapy for childhood tumors on the hypothalamic-pituitary-gonadal (HPG) axis. Finally, we will broadly review specific endocrine considerations regarding the therapies available for height preservation in this unique patient population.

## 2. Diagnosis of Gonadotropin-Dependent Precocious Puberty

Gonadotropin-dependent precocious puberty results from the premature activation of the HPG axis, which can occur directly from tumor involvement of the hypothalamus/pituitary or indirectly, such as seen with hydrocephalus (see below). The mechanisms that activate the HPG axis are poorly understood, but recent developments have contributed significantly to our understanding of pubertal onset and subsequent reproductive health. Among the most important recent discoveries has been the identification of kisspeptin, a ligand for the G-protein coupled receptor 54 [17–19]. The gene encoding kisspeptin (*Kiss1*) has been demonstrated to be mutated in some cases of hypogonadotropic hypogonadism [20, 21] and to be upregulated in some instances of precocious puberty [22–24]. It appears that kisspeptin expression is in part regulated by androgens and estrogens in a gender-specific manner [25]. Kisspeptin expression also appears to be influenced by leptin [26], which may help to explain the trend toward earlier pubertal onset among overweight youth. 

A careful history (including timing/extent of pubertal changes, family history, and associated symptoms such as headaches and visual loss) in addition to a comprehensive physical examination (including past and current growth velocity as well as a detailed assessment of sexual maturation) are essential [27]. Gender-specific changes, such as bilateral increase in testicular volume in boys and breast development in girls, may suggest gonadotropin-dependent pubertal development. However, it is important to realize that these findings may be variable depending on etiology and may also be found in gonadotropin-independent sexual precocity [28, 29]. Chalumeau et al. has identified three predictors of CNS lesions in girls, including age < 6 years, estradiol > 100 pmol/L, and absence of pubic hair [12]. Distinguishing pubertal variants such as benign premature thelarche, adrenarche, and menarche from precocious puberty is imperative so that significant pathology is not missed. 

A bone age (radiograph of the nondominant hand and wrist) is vital in the evaluation of sexual precocity, as it is expected to be advanced for chronologic age in cases of pathologic precocious puberty [30]. Skeletal age advancement in association with rapid progression of sexual maturation defines sexual precocity, but determining the exact etiology requires further evaluation. 

The diagnosis of gonadotropin-dependent precocious puberty is made by demonstrating a pubertal luteinizing hormone (LH) at baseline (specifically LH/follicle stimulating hormone (FSH) ratio > 0.2) [31] or in response to gonadotropin-releasing hormone (GnRH) or GnRH analog (GnRHa) stimulation [32–40]. The agent used, dosing, and route of administration (intravenous or subcutaneous) vary between studies, making it difficult to set exact cutoff values, so these tests should always be interpreted in the clinical context of the child being evaluated and the test being performed. 

At our center, our protocol is based upon that previously published by Garibaldi and colleagues [32], We draw baseline LH, FSH, and testosterone/estradiol levels (gender-dependent) followed by the subcutaneous administration of 20 micrograms/kg of leuprolide acetate (concentration of 1000 mcg/0.2 mL). LH and FSH samples are obtained at 60, 120, and 180 minutes after injection with testosterone/estradiol repeated at 180 minutes after injection. 

The use of ultrasensitive assays for measuring LH is of utmost importance in interpreting the data [41, 42], but the basal LH level may not always reflect pubertal stage secondary to the cyclic changes in gonadotropin secretion depending on pubertal status [43]. It has been reported that GnRH-stimulated LH levels greater than 4.1 IU/L (using ICMA) in boys and 3.3 IU/L (using ICMA) in girls are suggestive of precocious puberty [42]. However, in girls, there was significant overlap between prepubertal and pubertal values. Nevertheless, an LH-predominant response to exogenous GnRH or GnRHa is anticipated in the child with sexual precocity that is driven by premature activation of the HPG axis. 

If a pubertal LH level is demonstrated, brain magnetic resonance imaging (MRI) is generally indicated to look for a CNS lesion [44–46]. This is particularly true for very young patients (age ≤ 6) who present with gonadotropin-dependent precocious puberty and boys, noting that the recommendation for routine CNS imaging in older otherwise asymptomatic girls remains controversial [46–49]. 

Tumors that overproduce human chorionic gonadotropin (hCG) are also an important cause of precocious puberty in boys due to the cross-reaction of hCG with the LH receptor. Such tumors may be located centrally or peripherally (see further discussion below). Commonly, boys have less pronounced testicular enlargement secondary to lack of Sertoli cell stimulation from follicle stimulating hormone (FSH). Of importance, baseline and stimulated LH levels are prepubertal, but physical findings are consistent with gonadotropin-dependent precocious puberty. Measurement of serum *β*-hCG is the initial diagnostic test of choice, and assessing *β*-hCG levels in both serum and cerebrospinal fluid may help differentiate tumor location. In addition to brain MRI to look for pinealomas or dysgerminomas, it is also important to look for lesions in the mediastinum, liver, and gonads. The work-up of an hCG-secreting tumor should include a staged and symptom-oriented approach to imaging of the brain, chest, liver, and gonads. Cyclic surges and declines of *β*-hCG in such tumors have been described, making repeat measurements often necessary in suspect cases [50]. For the purposes of this review, such cases are categorized as gonadotropin-dependent precocious puberty because hCG is a gonadotropin and imparts a similar clinical presentation to gonadotropin-dependent precocious puberty.

## 3. Central Nervous System (CNS) Tumors

CNS tumors will be discussed in order of overall frequency of occurrence in childhood. Though CNS tumors are relatively common childhood neoplasms, tumors presenting with precocious puberty are relatively uncommon [51]. A number of CNS tumors contributing to precocious puberty have been described. Commonly, these tumors are located in the sellar and/or suprasellar region of the brain, thereby directly disrupting the normal prepubertal inhibition of the HPG axis. Sometimes tumors distant from the sella may indirectly cause GnRH stimulation through pressure on the hypothalamic-pituitary region from concurrent hydrocephalus [52]. Table 1 summarizes the potential causes of gonadotropin-dependent precocious puberty. 

**Table 1:** Causes of gonadotropin-dependent precocious puberty.

(i) Idiopathic
(ii) Central nervous system tumors (through direct or indirect effects on GnRH):
(1) Arachnoid cysts
(2) Craniopharyngiomas
(3) Ependymomas
(4) Germinomas (non-HCG secreting)
(5) Low-grade gliomas (juvenile pilocytic astrocytomas; optic pathway gliomas)
(iii) Paraneoplastic conditions (through the action of HCG on the LH receptor):
(1) Germ cell tumors:
(a) CNS
(b) Gonadal
(c) Hepatic
(d) Mediastinal (can occur in Klinefelter's syndrome)
(2) Hepatoblastoma
(iv) Developmental anomalies (through direct or indirect effects on GnRH):
(1) Arachnoid cysts
(2) Hydrocephalus
(3) Hypothalamic hamartomas
(v) Postirradiation (through direct effects on GnRH):
(1) Radiation therapy for childhood cancers (girls more susceptible)
(vi) Post-infectious, trauma, and bleed (through direct or indirect effects on GnRH):
(1) Sometimes associated with arachnoid cyst development

GnRH, gonadotropin releasing hormone; HCG, human chorionic gonadotropin; LH, luteinizing hormone.

Generally, CNS tumors are classified based on primary morphology and tumor location in children. Symptoms and signs of a primary CNS neoplasm depend on the growth rate of the tumor, its location, and age of the child [53]. Clinical findings can be quite varied, ranging from signs of increased intracranial pressure (ICP) to localizing neurological signs and symptoms. Weight loss, macrocephaly, and growth failure may also suggest the presence of a CNS tumor [51]. 

Infratentorial tumors are located in the posterior fossa and include medulloblastoma, cerebellar astrocytoma, brain stem glioma, ependymoma, and atypical teratoid rhabdoid tumors. These tumors commonly present with ataxia, cranial neuropathies, and signs of increased ICP, such as headaches and emesis. When precocious puberty presents in this setting, it is most likely secondary to increased ICP causing interference in the hypothalamic region. 

Supratentorial tumors include suprasellar tumors such as craniopharyngiomas, gliomas, germinomas, pineal tumors, supratentorial primitive neuroectodermal tumors, and ependymomas [53]. These tumors commonly present with visual disturbances and signs of increased ICP as well as possible neuroendocrine dysfunction. 

### 3.1. Low-Grade Gliomas

**Figure 1:**
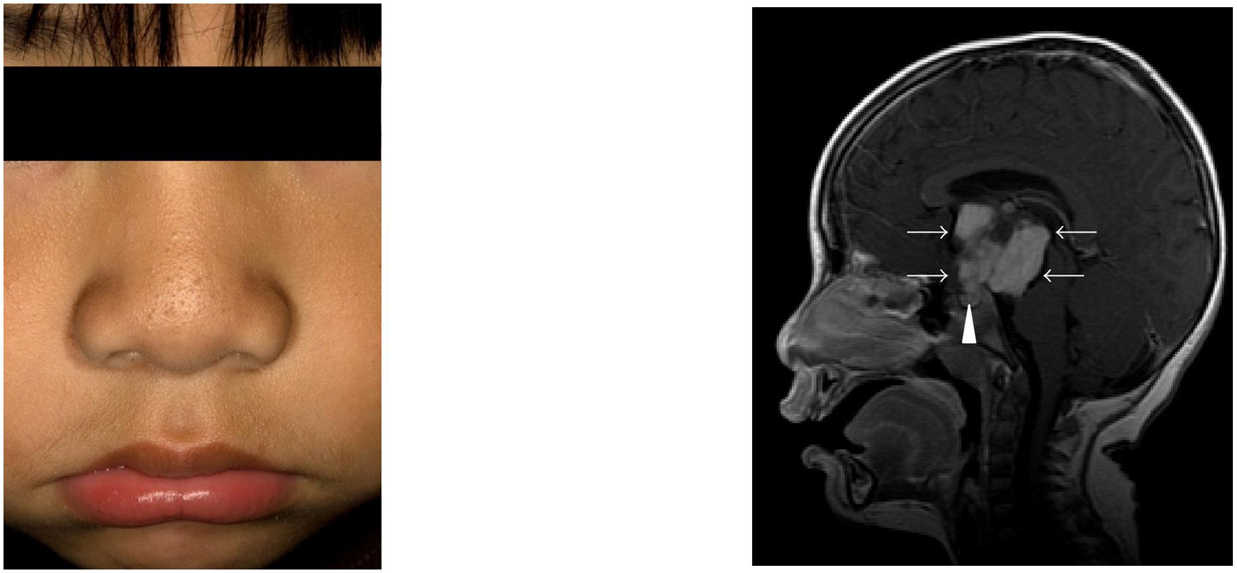
**(a) A 3-year-old male presented with Tanner II pubic hair, testicular enlargement (~6 mL bilaterally), facial hair, and acne.** Laboratory evaluation was consistent with gonadotropin-dependent sexual precocity. (b) MRI revealed a large suprasellar mass (arrows) with both solid and cystic components. The normal pituitary (arrowhead) is also visualized. Pathology confirmed a juvenile pilocytic astrocytoma.

Most low-grade gliomas (LGG) in children are juvenile pilocytic astrocytomas (JPA) or diffuse fibrillary astrocytomas, while oligodendrogliomas, oligoastrocytomas, and mixed gliomas are much less common. JPAs are most often found in the cerebellar region, though they may also be in other CNS regions including the hypothalamus/optic pathway or the spinal cord. They comprise approximately 50%–60% of CNS tumors, with greater than 75% occurring during childhood [55]. The average age of diagnosis is 6.5 to 9 years and boys are more commonly affected [56]. Though JPAs are generally well circumscribed and slow growing, this indolent growth pattern contributes significantly to their associated morbidities. Metastases are uncommon, although tumors in the hypothalamic and periventricular regions are more likely to spread. Commonly, children with LGG present with headache and seizure, though precocious puberty may be among the initial manifestations (Figure 1). 

**Figure 2:**
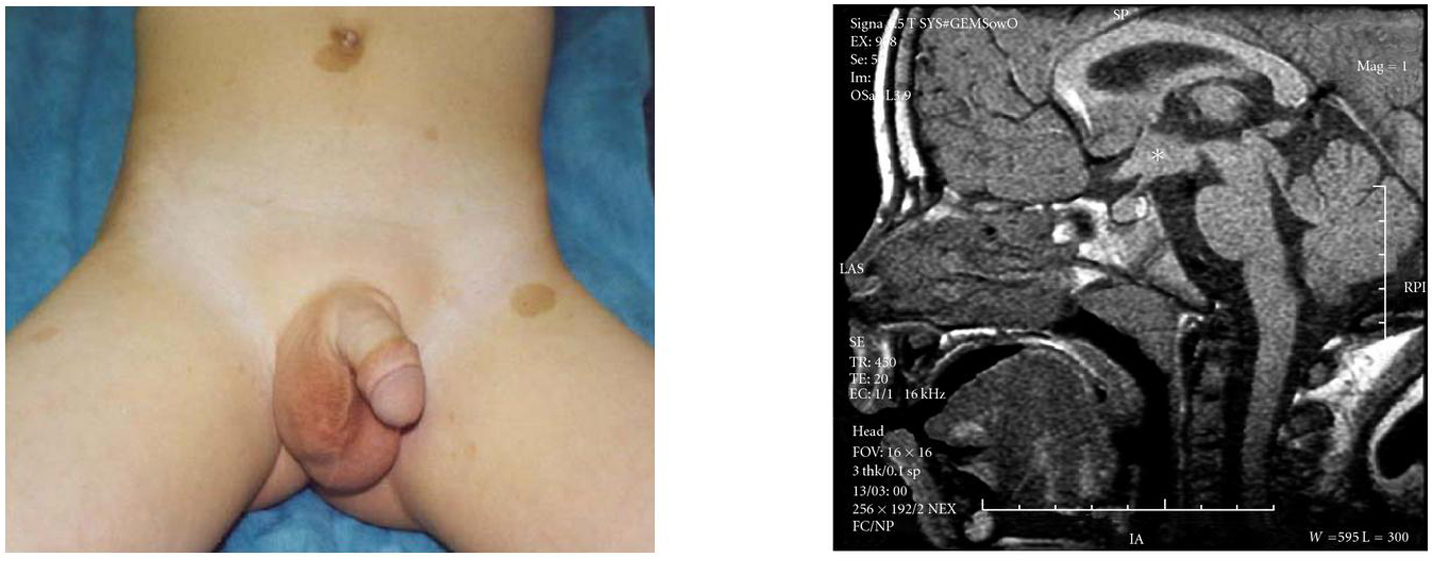
**(a) A 3-year-old male with neurofibromatosis type 1 (note classic café-au-lait macules) presented with a history of growth acceleration and testicular enlargement.** Bone age was advanced by 6 years. Gonadotropin-releasing hormone stimulation confirmed a diagnosis of gonadotropin-dependent precocious puberty, with a peak luteinizing hormone level of 20.9 mIU/mL. (b). MRI demonstrated a large optic pathway glioma (asterisk). (Figures obtained with permission [54].)

LGGs associated with the optic pathway are commonly found in patients with neurofibromatosis type 1 (NF-1). While at least 15% of patients with NF-1 develop optic pathway gliomas, approximately one-third of patients with optic pathway gliomas are subsequently found to have NF-1 [53]. NF-1 affects approximately one in 2500–3000 people [57–59]. It is an autosomal dominant neurocutaneous disorder with characteristic clinical findings, including café-au-lait macules with smooth borders (Figure 2), skinfold freckling, cutaneous neurofibromas, and iris hamartomas [60]. The clinical sequelae of NF-1 are due to inactivation of the tumor suppressor gene *neurofibromin-1*, which in turn normally inhibits the *Ras* gene, an important regulator of cell growth, differentiation, and survival [61, 62]. Upregulated *Ras* activity with or without a clear gene mutation may act in part through activation of the mTOR pathway [63–65]. Optic gliomas in association with NF-1 seem to contribute to precocious puberty through direct mass effect (Figure 2). The interested reader is referred to a recent comprehensive review of NF1 by Williams et al. [66].

The diagnosis of LGGs is confirmed through biopsy and histologic classification. Treatment of LGGs should be individualized depending on the location and clinical sequelae of the tumor, in addition to the overall clinical context (i.e., whether or not the child has NF1). With cerebellar JPAs, surgical resection is often curative. Generally, even those with incomplete resection have excellent long-term progression-free survival [53]. Chemotherapy is usually recommended for symptomatic or progressive tumors with the intention of delaying or avoiding radiotherapy. Further, it is recommended that surgical resection be reserved only for those with significant extension of the tumor, disfiguring proptosis, and/or rapid clinical deterioration [67].

### 3.2. Ependymomas

**Figure 3:**
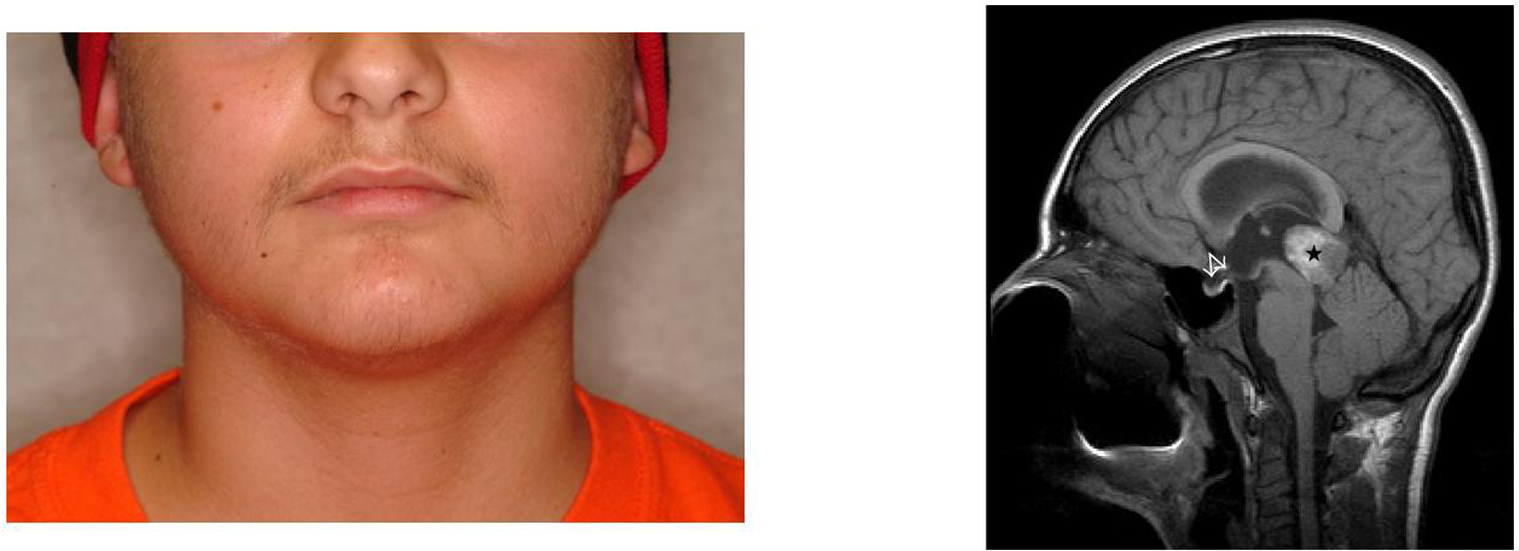
**(a) A 10-year-old male presented with significant facial and pubic hair growth, deepening voice, and minimal testicular enlargement (5 mL bilaterally).** Laboratory evaluation showed a markedly elevated *β*-hCG, pubertal testosterone, and suppressed gonadotropin levels, consistent with hCG-mediated sexual precocity. (b) MRI revealed a large pineal mass (star). Note the effects of tumor-induced hydrocephalus on the hypothalamic-pituitary unit (arrows). Pathology revealed a mixed germ cell tumor and the patient had a complete response to therapy. He entered endogenous puberty normally and has a final height of 68 inches (midparental height 71 inches).

Ependymomas tend to arise insidiously, and despite their predilection towards the lateral posterior fossa, they often cause obstructive hydrocephalus. Generally, these tumors are slow growing and well circumscribed. Ependymomas account for ~10% of CNS tumors in children [68]. The mean age at diagnosis is 3 years, with 50% being diagnosed prior to 5 years of age [69]. Boys are affected approximately 1.4 times more often than girls. Since greater than 70% of ependymomas arise from the posterior fossa, the signs at presentation are often a result of tumor-induced hydrocephalus [69]. This obstructive hydrocephalus may in turn lead to distinct effects on the hypothalamic region (Figure 3), including precocious pubertal onset. 

For ependymomas, total resection is the optimal therapy, which is more easily accomplished with supratentorial ependymomas [69]. The role of adjunct radiotherapy in children >3 years is well established, but it generally is not considered in younger children secondary to the potential effects of radiation on the developing brain [70]. Furthermore, ependymomas appear to be fairly resistant to chemotherapeutic regimens. However, there is renewed interest in using local radiotherapy in children as young as 1 year who have infratentorial tumors not amenable to surgical removal [71]. One review of prognostic factors shows that younger age appears to be the most important factor influencing survival [72].

### 3.3. Pineal Tumors

Pineal tumors include germ cell tumors, pineal parenchymal tumors, and glial tumors. These tumors comprise as much as 7% of CNS tumors in childhood [73]. The pineal gland is located adjacent to the brain stem and cerebral aqueduct, and tumors arising in this location may cause obstructive hydrocephalus (Figure 3). Loss of upward gaze (Parinaud's syndrome) may be seen secondary to brainstem compression. Dissemination is found in approximately 25% of patients at time of diagnosis [73]. Precocious puberty may occur with these tumors from either tumor-induced hydrocephalus or through gonadotropin secretion in the case of germ cell tumors (Figure 3; also see section on hCG-secreting tumors) [74, 75]. 

With pineal tumors, biopsy and histologic classification of tumor type is important prior to starting definitive therapy, because radiologic appearance alone will not define the type of pineal lesion present [73, 76]. The location of these tumors makes complete surgical resection quite difficult, necessitating adjunctive radiotherapy and chemotherapy, with variable long-term outcomes reported [53]. 

### 3.4. Craniopharyngioma

Craniopharyngiomas are slowly growing tumors of the sellar region with insidious onset [77, 78]. At the time of diagnosis, most patients have both neurologic and endocrine signs and symptoms related to disruption of hypothalamic-pituitary function and increased ICP/mass effect [77, 78]. These tumors account for 5% of CNS tumors and the majority of sellar tumors diagnosed in childhood [79]. They have a bimodal distribution with peak incidences from 5–14 years and again from 65–74 years of age [78, 80–82]. While the endocrine manifestations usually involve varying degrees of hypopituitarism, precocious puberty may also occur [83, 84]. The growth spurt typically expected with precocious puberty may be masked by concomitant growth hormone deficiency [84]. Computed tomography is helpful to identify the pathognomonic calcification that is a radiologic hallmark of craniopharyngioma, but MRI is preferred secondary to its superiority in detailing anatomy and tumor extent [77, 78]. 

Total surgical resection of craniopharyngiomas is associated with significant morbidity (including but not limited to hypothalamic obesity, panhypopituitarism, and altered neuropsychological profile) and mortality risk (up to 10%) [85–87]. Recurrence, even with complete resection, occurs in as many as 15% of these patients [78] and is associated with an even higher morbidity and mortality risk [88, 89]. Selective debulking along with adjunctive radiotherapy may be a more appropriate approach in these children [85].

## 4. Other Central Nervous System Lesions

### 4.1. Hypothalamic Hamartomas

**Figure 4:**
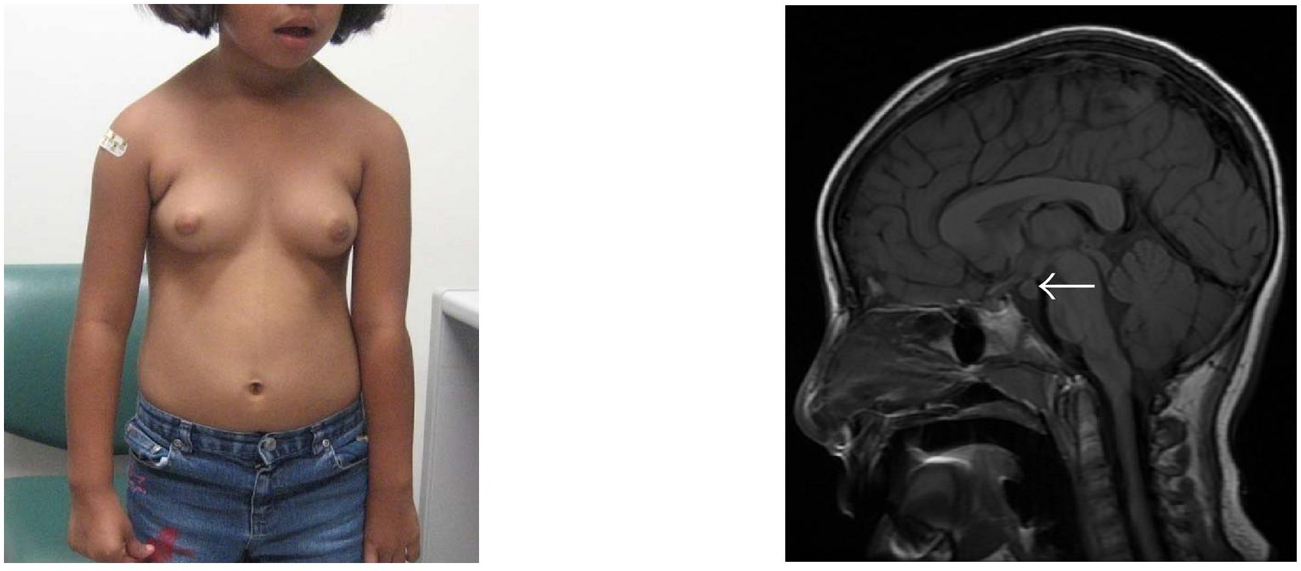
**(a) A 4-year-old female presented with Tanner III-IV breast development and bone age advancement to 11 years of age.** Leuprolide stimulation (stimulated luteinizing hormone of 28 mIu/mL) confirmed gonadotropin-dependent precocious puberty. (b) MRI revealed an isointense mass (arrow) consistent with the diagnosis of a pedunculated hypothalamic hamartoma. This young girl's puberty has been adequately suppressed with depot leuprolide without further bone age advancement, pubertal development, neurologic sequelae, or mass changes on serial MRIs.

Hypothalamic hamartomas are nonneoplastic developmental lesions that are usually histologically normal in appearance, but ectopic in position [90]. They are composed of heterotopic grey matter, neurons, and glial cells usually located at the base of the third ventricle, near the tuber cinereum or mammillary bodies. Hypothalamic hamartomas have a typical isointense radiographic appearance on MRI (Figure 4). They are classified as pedunculated or sessile, depending on the width of attachment to the tuber cinereum and their pattern of growth, namely intra- or extraparenchymal [91, 92]. These lesions are believed to cause precocious puberty (Figure 4) through endogenous pulsatile release of GnRH, either independently or in concert with the GnRH-secreting neurons of the hypothalamus [93]. It has also been suggested that precocious puberty may be caused through the indirect actions of glial factors, including transforming growth factor alpha, that stimulate GnRH secretion from the hypothalamus [94, 95]. Removal of the hamartoma does not prevent or inhibit further pubertal development in some patients. In these patients, secondary activation of astroglial cells in the surrounding hypothalamic tissue may cause increased GnRH secretion, thereby inducing precocious puberty [94–96]. 

In patients with hamartomas, the classic triad of precocious puberty, developmental delay, and seizures, most notably gelastic ("laughing") seizures, is well described. Patients with hypothalamic hamartoma usually present at <4 years of age with precocious puberty [97, 98]. Precocious puberty is found in 33%–85% of patients with a hypothalamic hamartoma, many of whom also develop seizures [99]. It is a rare condition with a prevalence from 1 : 50,000–100,000 [100]. Pedunculated hamartomas are more likely to be associated with precocious puberty while sessile hamartomas are more likely to be associated with seizures [100, 101]. Generally, the presentation is more severe in younger patients and tends to progress towards a debilitating seizure disorder with marked developmental delay, while older patients tend to have a less severe seizure disorder and less developmental impairment. 

Hypothalamic hamartomas are typically sporadic but may also be associated with the Pallister Hall Syndrome (PHS) [102]. PHS is an autosomal dominant syndrome with anomalies including hypothalamic hamartoma, pituitary abnormalities (including aplasia/dysplasia and/or hypopituitarism), imperforate anus, and polydactyly [103, 104]. PHS is due to mutations of the zinc-finger transcription factor gene *GLI3* on chromosome 7p13. *GLI3* has been demonstrated to have a role in sonic hedgehog-mediated brain development. Disruption of this gene or associated genes may explain some cases of hypothalamic hamartoma [105]. A number of other candidate genes are being investigated for potential roles in hypothalamic hamartoma formation and its clinical sequelae [106]. 

Treatment of hypothalamic hamartomas varies depending upon the patient's symptoms and appearance of the tumor. Generally, precocious puberty can be adequately treated with GnRH analog therapy, whereas treating the associated seizures can be much more challenging [107]. Téllez-Zenteno et al. have recently reviewed the various surgical and nonsurgical approaches to the treatment of hypothalamic hamartomas [108]. The transcallosal surgical approach has shown to be the most effective for seizure control, although a number of other therapies, including stereotactic radiosurgery, have shown promise [108].

### 4.2. Arachnoid Cysts

Arachnoid cysts are relatively uncommon intracranial lesions, usually developmental in origin, but they may also develop after infection, trauma, or hemorrhage [109]. Furthermore, arachnoid cysts have also been described in association with hypothalamic hamartomas and tuberous sclerosis [110, 111]. Neurologic and visual field disturbances are common. Arachnoid cysts are also commonly associated with other midline defects and optic nerve hypoplasia [109]. When located in the suprasellar region, endocrine findings are common, including gonadotropin-dependent precocious puberty. A number of cases have reported precocious puberty along with pituitary hormone deficits as well as neurologic findings, specifically the bobble-head doll phenomenon (rhythmic to-and-fro bobbling of the head and trunk) [112–115]. Interestingly, delayed puberty has also been reported with arachnoid cysts and other suprasellar lesions [116, 117]. Arachnoid cysts can be successfully managed with stereotactic ventriculocystostomy [113, 118].

### 4.3. hCG-Producing Tumors

**Figure 5:**
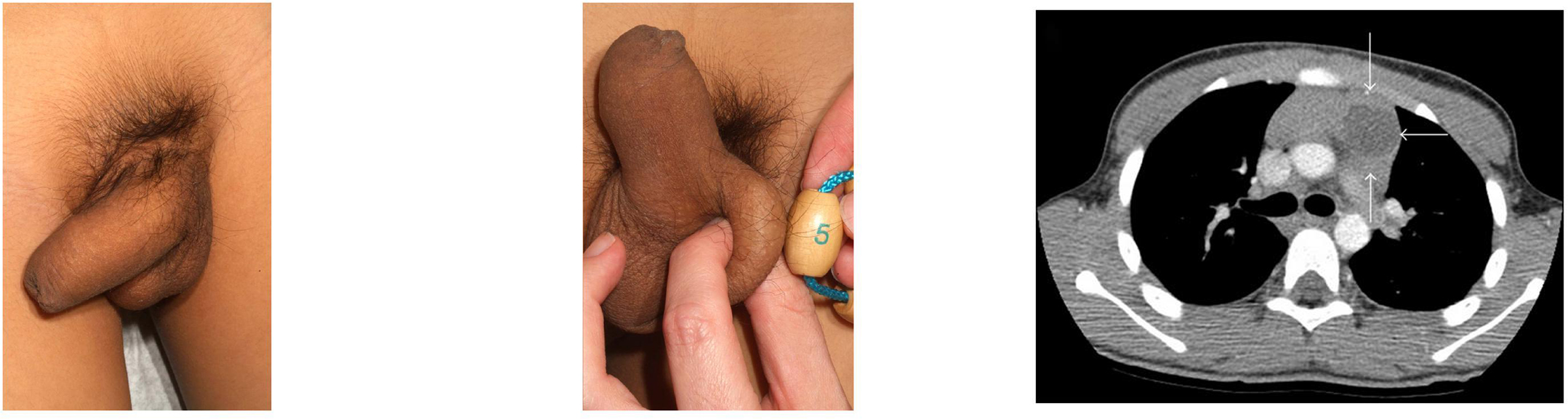
**A 4-year-old male with a history of asthma presented with complaints of pubertal changes (pubic hair growth, erections, sexual behaviors, acne, deepening of the voice, accelerated linear growth, and increased muscle mass).** (a) On examination, he had an enlarged penis and Tanner III-IV pubic hair; testes were minimally enlarged (b) Bone age was 10 years and laboratory evaluation revealed a total testosterone of 673 ng/dL (normal ≤5), *β*-hCG of 22.9 mIU/mL (normal ≤1.0), and undetectable gonadotropin levels, consistent with hCG-mediated sexual precocity. (c) CT chest revealed a  cm heterogeneous mass located in the anterior mediastinum (arrows). This lesion was resected and confirmed to be a mature cystic teratoma. After surgery, the patient's labs normalized, and he remains clinically prepubertal at a chronological age of 9 years and bone age of 13 years.

Germ cell tumors can produce excessive hCG, which causes precocious puberty, almost exclusively in males, via cross-reaction with the LH receptor. Typically arising from the brain (Figure 3), mediastinum (Figure 5), or gonads, these tumors are characterized based on histologic differences. Regardless of locale, the child may present with manifestations typical of isosexual precocious puberty, but with suppressed basal and/or stimulated LH levels. Girls with hCG-secreting tumors uncommonly present with precocious puberty secondary to the requirement for both LH and FSH effects at the ovary for pubertal onset [119]. Intracranial hCG-secreting tumors may present with isosexual precocious puberty, irrespective of gender, secondary to disinhibition of GnRH, weak FSH effect (as a consequence of massive hCG secretion), and cosecretion of estradiol by some dysgerminomas [120, 121]. There is a well-recognized association of mediastinal hCG-secreting germ cell tumors in patients with Klinefelter's syndrome [122, 123].

## 5. Radiation Related Precocious Puberty

Cranial irradiation is a commonly used modality for the treatment of primary CNS tumors, and it can also be used in the adjunctive treatment of other childhood malignancies. The effects of radiotherapy on the hypothalamic-pituitary axis are variable and may evolve over a prolonged period of time. Somatotrophs are the anterior pituitary cell type most sensitive to irradiation of the hypothalamic-pituitary axis followed by the gonadotrophs, corticotrophs, and thyrotrophs [124]. 

**Figure 6:**
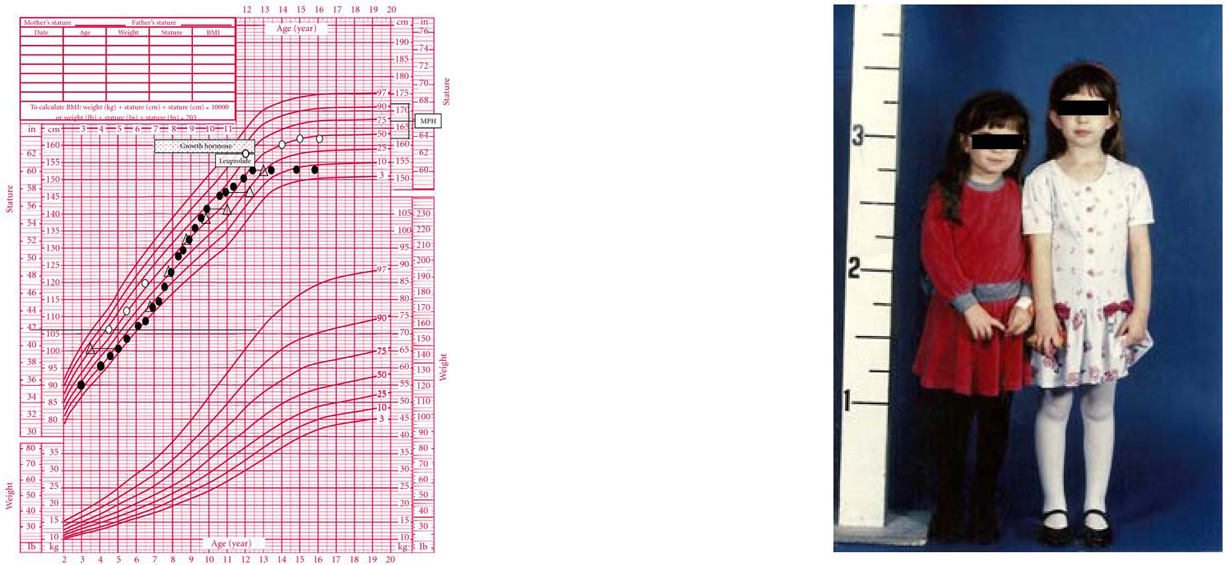
**(a) Growth chart of a girl (solid circles) with a history of locally advanced retinoblastoma status post right eye enucleation, chemotherapy, and radiotherapy (39.**6 Gy in 35 fractions) to the orbit, completed at the age of 17 months. Bone ages are represented by open triangles. She was diagnosed with growth hormone (GH) deficiency and then gonadotropin-dependent sexual precocity and treated with GH and depot leuprolide, respectively. The patient had menarche at age 8 and achieved a final height of 60 inches, well below mid-parental height (MPH). (b) The patient's identical twin sister (open circles) was already significantly taller by the age of 5 years. She had menarche at age 12 and achieved a final adult height of 63.75 inches.

Lower doses (18–24 Gray) of radiotherapy are often associated with precocious puberty in girls [125–127], whereas doses higher than 25 Gray can affect both sexes, with younger age at radiotherapy conferring a higher risk of precocious puberty (Figure 6) [127–129]. Furthermore, patients who develop precocious puberty following doses of 30 Gray or more have a significant risk of ultimately developing gonadotropin deficiency, while those who receive doses in excess of 50 Gray are at increased risk of delayed puberty (secondary to gonadotropin deficiency) [130–132]. 

A recent report from the Childhood Cancer Survivor Study found a significantly increased risk of both early and late menarche in cancer survivors when compared with their siblings [133]. In this study, radiotherapy >50 Gray and age of treatment ≤4 years conferred a higher risk for early menarche. While lower doses of radiotherapy also increased the odds of early menarche, statistical significance was not demonstrated. Another report of female acute lymphoblastic leukemia (ALL) survivors demonstrated that those who were treated with cranial radiotherapy had higher rates of early menarche and those treated with craniospinal radiotherapy had higher rates of both early and late menarche when compared to those treated with chemotherapy alone [134]. This study also found an increased risk of early menarche among those diagnosed at <5 years of age. 

In childhood cancer survivors, the presentation of sexual precocity can be subtle, the definitions of normal puberty may not apply, and the diagnosis may not be straightforward. For example, patients who develop precocious puberty after irradiation may also have concomitant growth hormone deficiency, which in turn can mask pubertal growth acceleration and bone age advance, in turn delaying the diagnosis of sexual precocity (Figure 6) [135, 136]. Importantly, testicular volume in boys who are childhood cancer survivors may not be reliable in the identification of pubertal onset, and so a high index of suspicion and measurement of gonadotropin and testosterone levels in boys previously treated with radiation and chemotherapy is paramount [137, 138].

## 6. Treatment of Gonadotropin-Dependent Precocious Puberty-Endocrine Considerations

The appropriate treatment of gonadotropin-dependent precocious puberty is initially contingent on correctly identifying the etiology. GnRHa therapy has been demonstrated to be quite effective at stalling puberty and preserving adult height (particularly when started at <6 years of age) in children with precocious puberty due to premature activation of the HPG axis [139]. In children with hCG-secreting tumors, the precocious puberty is best addressed by treating the underlying tumor, although the natural history of endogenous puberty in such children, who in our experience tend to have markedly advanced skeletal maturation, remains poorly characterized. 

The treatment of brain lesions contributing to precocious puberty is tumor dependent and is also dependent on a number of other factors, including age, comorbidities, and location of the tumor. Importantly, the endocrinologist should carefully evaluate the remainder of the hypothalamic-pituitary axis prior to definitive therapy and regularly after therapy is complete [140]. Treatment of pituitary hormone deficiencies should be undertaken as clinically indicated, recognizing that the diagnosis of clinically important endocrinopathies, such as central hypothyroidism, may be difficult to make in this population.

Children diagnosed with a brain tumor prior to 4 years of age and those who receive radiation potentially affecting the hypothalamic-pituitary axis are at highest risk of adult short stature [141]. Helping such children who develop sexual precocity achieve a normal adult height may be difficult, and multimodality hormonal therapy may need to be considered. Commonly, these children are not diagnosed until a later age and/or may have such an advancement of skeletal maturity that GnRHa therapy alone may not salvage adult height. In these situations, the use of growth hormone and/or aromatase inhibitor therapy may be considered but remains largely unstudied in this population. The addition of growth hormone to GnRHa therapy has shown variable responses in height gain dependent on duration of therapy [142–144]. Among children who received spinal radiation therapy, age at treatment appears to influence adult height most, and boys seem to be less responsive to growth hormone therapy than girls [145]. Aromatase inhibitors have been shown to increase predicted adult height in normal boys treated with growth hormone while allowing normal pubertal progression [146–148]. Our initial clinical experience has demonstrated an increased predicted adult height with the use of growth hormone and/or aromatase inhibitors, but this is an area that needs to be researched further via prospective clinical trials in terms of long-term safety and efficacy.

## 7. Conclusion

When evaluating children with precocious puberty, possible neoplastic, developmental, and iatrogenic causes should be considered in the differential diagnosis, particularly in boys and in childhood cancer survivors. Through prompt evaluation and treatment, long-term sequelae, specifically short stature and possible impaired quality of life, may be avoided. A heightened awareness of the neoplastic causes of gonadotropin-dependent precocious puberty and vigilance in the evaluation of children presenting with precocious puberty are of utmost importance in order to avoid missing important pathology.

## Conflicts of Interests

The authors have nothing to disclose.

## Abbreviations

LH: Luteinizing hormone

*β*-HCG: Beta-human chorionic gonadotropin

GnRH: Gonadotropin releasing hormone

PHS: Pallister-Hall Syndrome

ICP: Intracranial pressure

LGG: Low-grade glioma

JPA: Juvenile pilocytic astrocytoma

NF-1: Neurofibromatosis type 1

TSH: Thyroid stimulating hormone

ACTH: Adrenocorticotropin hormone

AVP: Arginine Vasopressin

HPG: Hypothalamic-pituitary-gonadal

MRI: Magnetic resonance imaging.
